# A Change in the Air: Smoking Bans Gain Momentum Worldwide

**DOI:** 10.1289/ehp.115-a412

**Published:** 2007-08

**Authors:** Charles W. Schmidt

On 29 March 2004, Ireland became the first country in the world to ban smoking in all indoor workplaces, including in restaurants and bars. That landmark event followed a ten-year period during which it was shown that voluntary bans and partial bans allowing workplace smoking in certain designated areas didn’t protect Irish workers from secondhand smoke, a known cause of cancer and other lethal diseases. So in a move to protect its workers, Ireland took the extraordinary step of banning indoor workplace smoking completely. In so doing, it launched a wave of similar national-level policies now spreading across the globe.

## The Ripple Effect

Since 2004, countries including Norway, New Zealand, Uruguay, Malta, Italy, Sweden, Scotland, Bhutan, Lithuania, and the British Virgin Islands have gone smoke-free, protecting the health of millions by banning smoking in public places. The European Union is now considering smoke-free legislation for all of its 27 member countries. France—the latest country to move in this direction—issued a workplace ban in January 2007, which will apply to restaurants and bars next year. Other countries and territories now preparing to adopt national bans include England, Wales, Northern Ireland, Kazakhstan, Finland, and Hong Kong. Most of Canada and Australia is covered by smoke-free policies.

Ireland didn’t spawn the trend in isolation. Beginning in the early 1980s, U.S. cities and towns began enacting ordinances that restricted, but did not eliminate, smoking in public places, workplaces, and restaurants. However, as understanding of the health effects of secondhand smoke grew, especially as a matter of worker protection, ordinances more commonly completely prohibited smoking, and expanded the locations covered by the law.

By 2004, entire states and municipalities in Canada, the United States, and Australia had adopted smoke-free laws, according to Americans for Nonsmokers’ Rights, a nonprofit advocacy group in Berkeley, California. Ten U.S. states, for instance, already had some form of smoke-free legislation, notably California, which had issued a ban in restaurants and bars by 1998, and Massachusetts, which in 2004 adopted an indoor workplace ban that included restaurants and bars. Since then, seven more U.S. states have imposed comprehensive workplace bans covering bars and restaurants, and ten more states are preparing to do the same, according to Americans for Nonsmokers’ Rights.

Ireland’s policy was significant because it showed a workplace ban could succeed in an entire country. “We had officials from [throughout Europe] come here so they could see what we had done with their own eyes,” says Luke Clancy, director general of the independent Research Institute for a Tobacco Free Society in Dublin, and the person who spearheaded the Irish campaign. “How much influence we had is perhaps best known to those who asked for our advice,” he says. “But they did ask, and now many have done the same.”

## A Tipping Point for Health

Greg Connolly, a professor in public health practice at the Harvard School of Public Health (HSPH), who pioneered the Massachusetts ban while heading the state’s tobacco control program, says a global smoke-free movement is under way, and likely past a tipping point. “The world’s begun to reclaim clean [indoor] air as a social norm,” he says.

What’s driving the trend? Experts point to a number of factors, including a growing awareness of the carcinogenic and other health risks of secondhand smoke. The WHO’s 2004 Framework Convention on Tobacco Control (FCTC) also has played a key role, says Damon Moglen, vice president for international programs with the Campaign for Tobacco-Free Kids, a Washington, DC–based nonprofit organization.

That treaty, now ratified by 145 countries, stipulates numerous provisions for its members, among them to eliminate tobacco advertising, to enhance warning labels on tobacco products, to establish clean indoor air laws, and to clamp down on tobacco smuggling, which involves some 6–8.5% of the 5.5 trillion cigarettes produced every year worldwide, according to the 2000 World Bank/WHO report *Tobacco Control in Developing Countries*. “Countries that ratified the FCTC have obligated themselves to bring about tobacco control measures,” Moglen says. “Many of the new European measures were put in place after the FCTC was enacted.”

On perhaps a more practical level, Ireland and Massachusetts added to the body of research showing that workplace bans could be enforced without economically burdening the hospitality industry, which had been a key rallying point for the tobacco industry. In an article published in the 12 April 2007 *New England Journal of Medicine*, Connolly and colleague Howard Koh, an HSPH professor of public health practice, wrote that “more than 20 high-quality studies have shown no negative economic effect of smoke-free policies on restaurants and bars.”

Finally, mounting evidence shows that comprehensive smoking bans produce real health benefits. In Ireland, indoor air contaminants in pubs have fallen dramatically since the ban came into force, according to a study by Clancy and colleagues that was published in the 15 April 2007 issue of the *American Journal of Respiratory and Critical Care Medicine*. From just prior to the ban to a year later, there was an 83% reduction in fine particulate matter and an 80.2% reduction in benzene concentrations in the pubs, along with a 79% reduction in exhaled breath carbon monoxide and an 81% reduction in salivary cotinine among nonsmoking pub workers. After the ban, the workers also showed statistically significant improvements in measured pulmonary function tests and far fewer self-reported respiratory and upper airway symptoms.

In Massachusetts, the ban may have the potential to accelerate a drop in cigarette sales, which are now half what they were when the state began its comprehensive tobacco control program in 1993. Connolly says rising cigarette taxes account for one-third of the drop in tobacco consumption—an article by Kenneth Warner, a professor at the University of Michigan School of Public Health that was published in the June 2005 issue of the *American Journal of Public Health* showed that for every 10% rise in cigarette taxes, sales drop by 8%. The rest of the drop is attributed to efforts including public smoking bans, mass media messages, smoking cessation services, and enforcement of laws preventing youth access to tobacco products.

## The Shift to Developing Countries

Now, with growing awareness of smoking risks, coupled with smoke-free legislation, higher rates of tobacco taxation, and numerous other measures, tobacco use is falling in the developed world. In the United States, decreased cigarette smoking is a major factor underlying the 40% decrease in cancer mortality rates among U.S. men over the past decade, states a report in the October 2006 issue of *Tobacco Control*.

The multinational tobacco industry is now turning increasingly to developing countries for growth. Of the world’s 1.3 billion smokers, 900 million live in developing and transitional economy nations, according to a February 2006 WHO fact sheet.

Connolly says tobacco firms exploit opportunities in poor countries that aren’t available elsewhere; they buy off corrupt officials to create favorable conditions for importing foreign cigarettes, they insinuate themselves into local economies, and they use advertising methods long since outlawed in the West. For instance, billboards hawking tobacco products are often found clustered near schools and playgrounds in developing nations, offering clear evidence of the industry’s efforts to lure children, Connolly says.

Tobacco advertisers in poor countries also specifically target women, adds Moglen. “They use messages that were common in the United States twenty years ago,” he says. “It’s the old Virginia Slims approach—they are erroneously suggesting that independence and allure can be attained by smoking.” To put these efforts in context, only 9% of women in developing nations smoke, compared with 49% of men, says Michele Bloch, a medical officer with the National Cancer Institute’s Tobacco Control Research Branch.

At the same time, multinational tobacco firms are basing production facilities directly in developing countries. Philip Morris, for example, recently launched a joint venture with the China National Tobacco Company, a monopoly that supplies 1.7 trillion cigarettes to China’s 350 million smokers annually. The deal allows Philip Morris to make cheaper Marlboros in China, and gives its Chinese partner access to global distribution networks.

Connolly’s view is that antismoking campaigns in these countries need to fight fire with fire. Just as tobacco firms used media to propagate the macho stereotype of the now defunct Marlboro Man, he explains, health officials need media to personalize smoking’s true risk. To that end, Connolly, Koh, and their collaborators use local research to reveal smoking’s community impacts both domestically and abroad.

Their investigations often yield alarming local data, such as high levels of cigarette-derived indoor air pollution, extensive child-oriented tobacco advertising, high numbers of expected deaths from smoking in a given community, and childhood risks from secondhand smoke exposure. Armed with that information, they use press conferences and counter-advertising to put smoking threats in a local context.

Connolly plans to use that approach now in the Mediterranean island nation of Cyprus, where 38% of the men and 10% of the women smoke. Through a joint effort coordinated by HSPH and the national government, Connolly and Philip Demokritou, a Cypriot national and HSPH faculty member, plan to rely heavily on local research to develop countermarket advertising, restrictions on youth access to tobacco products, and smoking cessation treatment programs, among others. Their proposed measures are described in the 2006 document *A Strategic Plan for Tobacco Control in Cyprus*.

## A Cultural Approach

In his June 2005 *American Journal of Public Health* article, Warner wrote that researchers need to consider carefully how they convey smoking risks to people who may not worry about tobacco’s more long-term consequences, particularly when faced with more immediate threats such as poverty, war, and infectious disease.

“In many places,” adds Moglen, “people don’t understand the scope and range of public health problems related to smoking.” Along those lines, researchers know that cigarette warning labels that display graphic pictures of mouth and lung cancer are effective smoking deterrents. Connolly and others have argued for their universal use, but the tobacco industry has fought with equal ferocity for verbal warnings, which have lesser impacts.

In short, the war against Big Tobacco has made key strides in many developed countries, where tobacco use is decreasing, and more and more people enjoy legal protection from secondhand smoke. But that battle is just getting under way in developing countries, where untold numbers face growing risks. Smoking is expected to kill 1 billion in the twenty-first century, according to the 2006 WHO *Tobacco Atlas*. Ideally, those working to limit smoking will drum up the sustainable momentum that’s needed to stop that trend in its tracks.

## Figures and Tables

**Figure:**
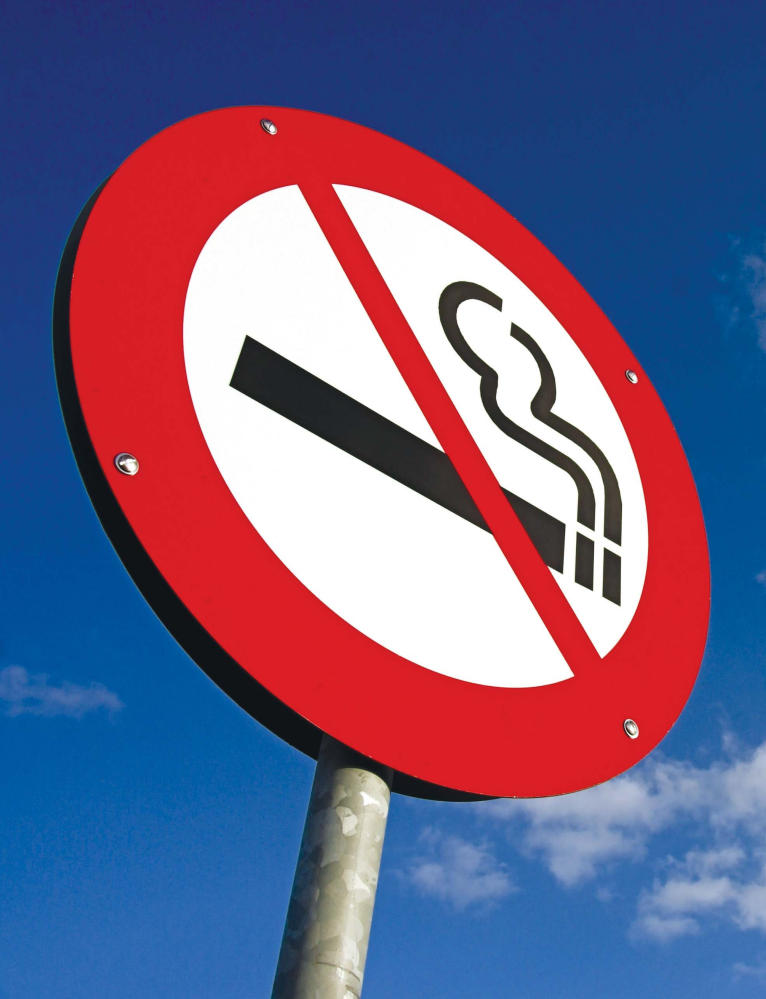


**Figure:**
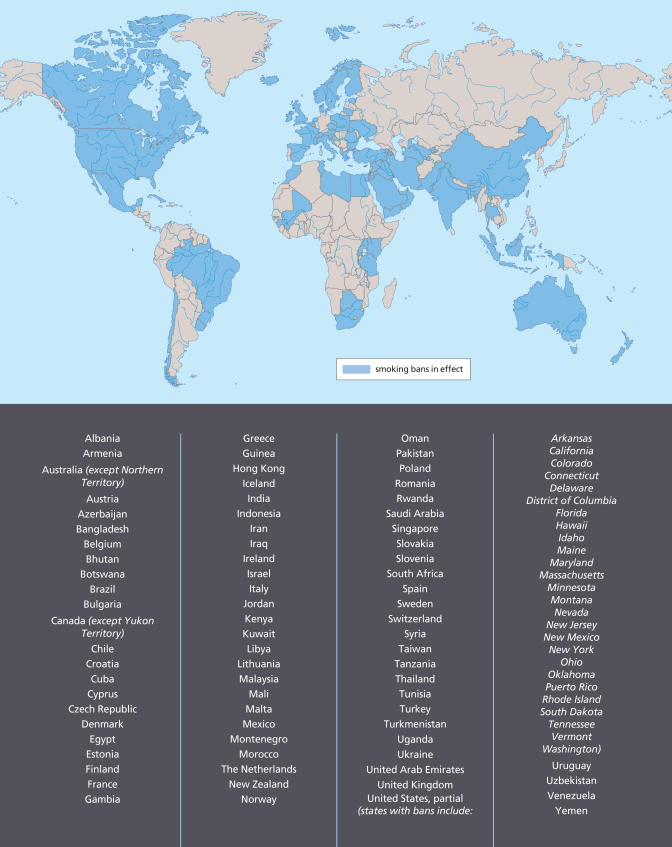
Smoking Bans Around the World

